# Assessing the Impact of Spatial Resolution on the Estimation of Leaf Nitrogen Concentration Over the Full Season of Paddy Rice Using Near-Surface Imaging Spectroscopy Data

**DOI:** 10.3389/fpls.2018.00964

**Published:** 2018-07-05

**Authors:** Kai Zhou, Tao Cheng, Yan Zhu, Weixing Cao, Susan L. Ustin, Hengbiao Zheng, Xia Yao, Yongchao Tian

**Affiliations:** ^1^National Engineering and Technology Center for Information Agriculture, Key Laboratory for Crop System Analysis and Decision Making, Ministry of Agriculture, Jiangsu Key Laboratory for Information Agriculture, Jiangsu Collaborative Innovation Center for Modern Crop Production, Nanjing Agricultural University, Nanjing, China; ^2^Center for Spatial Technologies and Remote Sensing, Department of Land, Air, and Water Resources, University of California, Davis, Davis, CA, United States

**Keywords:** leaf nitrogen concentration (LNC), imaging spectrometers, spatial resolutions, paddy rice, vegetation indices (VIs), Gaussian Process Regression (GPR), Partial Least Squares Regression (PLSR)

## Abstract

Timely monitoring nitrogen status of rice crops with remote sensing can help us optimize nitrogen fertilizer management and reduce environmental pollution. Recently, the use of near-surface imaging spectroscopy is emerging as a promising technology that can collect hyperspectral images with spatial resolutions ranging from millimeters to decimeters. The spatial resolution is crucial for the efficiency in the image sampling across rice plants and the separation of leaf signals from the background. However, the optimal spatial resolution of such images for monitoring the leaf nitrogen concentration (LNC) in rice crops remains unclear. To assess the impact of spatial resolution on the estimation of rice LNC, we collected ground-based hyperspectral images throughout the entire growing season over 2 consecutive years and generated ten sets of images with spatial resolutions ranging from 1.3 to 450 mm. These images were used to determine the sensitivity of LNC prediction to spatial resolution with three groups of vegetation indices (VIs) and two multivariate methods Gaussian Process regression (GPR) and Partial least squares regression (PLSR). The reflectance spectra of sunlit-, shaded-, and all-leaf leaf pixels separated from background pixels at each spatial resolution were used to predict LNC with VIs, GPR and PLSR, respectively. The results demonstrated all-leaf pixels generally exhibited more stable performance than sunlit- and shaded-leaf pixels regardless of estimation approaches. The predictions of LNC required stage-specific LNC~VI models for each vegetative stage but could be performed with a single model for all the reproductive stages. Specifically, most VIs achieved stable performances from all the resolutions finer than 14 mm for the early tillering stage but from all the resolutions finer than 56 mm for the other stages. In contrast, the global models for the prediction of LNC across the entire growing season were successfully established with the approaches of GPR or PLSR. In particular, GPR generally exhibited the best prediction of LNC with the optimal spatial resolution being found at 28 mm. These findings represent significant advances in the application of ground-based imaging spectroscopy as a promising approach to crop monitoring and understanding the effects of spatial resolution on the estimation of rice LNC.

## Introduction

Nitrogen (N) is the key nutrient parameter determining the photosynthetic functioning and productivity in crops (Inoue et al., 2012). N application deficiency could lead to lower chlorophyll content, lower photosynthetic assimilation, less biomass production, and reduced grain yield (Wang et al., 2014; Jay et al., 2017). Higher N application can improve the crop yield but it would also cause a series of environmental pollution issues and even yield decrease when the fertilization becomes excessive (Kaushal et al., 2011; Inoue et al., 2012). Therefore, timely monitoring of crop N status for precision N management purposes is critical for increasing grain yields and N use efficiency while also reducing environmental pollution (Miao et al., 2011; Zhang et al., 2011; Inoue et al., 2012).

In the past few decades, remote sensing has been proven as a promising approach to estimate crop N status at the field scale. As one of the most important crops for global food security, rice (*Oryza sativa* L.) has been investigated in numerous studies for determining canopy leaf N concentration (LNC) or plant N concentration using canopy reflectance spectra collected with field spectrometers (e.g., ASD FieldSpec Pro spectrometer) (Table 1). A series of studies applied multivariate regression (e.g., stepwise multiple linear regression) in the selection of optimal bands or variables for detecting rice N status (e.g., Tang et al., 2007; Yu et al., 2013) (Table 1). These methods usually produce high predictive accuracy but sometimes at the cost of over-fitting and intensive computation, especially when excessive variables were selected (Yu et al., 2013). Other studies use vegetation indices (VIs) that employ two or three bands in the visible, red-edge, near-infrared (NIR), or shortwave infrared regions to assess rice N status (e.g., Xue et al., 2004; Wang et al., 2012; Tian et al., 2014). These VI-based approaches are easier to operate as compared to those multivariate regressions and can also produce higher accuracies and better robustness in assessing N status.

**Table 1:** Summary of studies on the spectroscopic estimation of rice N status using field spectrometer measurements.

**Reference**	**Coverage of growth stages**	**Method**	**Data source**	**Nitrogen concentration**
Xue et al., 2004	Tillering, jointing, heading, and filling	SR(810, 560)	MSR16 radiometer	Leaf N
Nguyen and Lee, 2006	Panicle initiation and booting	PLS model	GER 1500 spectroradiometer	Leaf N
Tang et al., 2007	Booting, heading, milking, and maturing	SMLR model	FieldSpec Pro FR spectroradiometer	Leaf N
Zhu et al., 2007a	Jointing, booting, heading, and filling	NDVI(1220, 710)	MSR16 radiometer	Leaf N
Zhu et al., 2007b	Heading and filling	NDVI(1220, 610)	MSR16 radiometer	Leaf N
Lee et al., 2008	Panicle initiation	D_735_	Model LI-1800 spectroradiometer	Plant N
Stroppiana et al., 2009	Tillering, stem elongation, booting, flowering	NDVI(503, 483)	FieldSpec Pro FR spectroradiometer	Plant N
Wang et al., 2012	Jointing, booting, heading, and filling	(R_924_ –R_703_ + 2 × R_423_)/(R924 + R703 −2 × R423)	FieldSpec Pro FR spectroradiometer	Leaf N
Yu et al., 2013	Tillering, jointing, booting, heading, flowering, and filling	SMLR model	FieldSpec Pro FR spectroradiometer	Leaf N
Cao et al., 2013	Panicle initiation, stem elongation, and heading	SMLR model	Crop Circle ACS-470 active sensor	Plant N
Tian et al., 2014	Tillering, jointing, booting, heading, filling and milking	SR(553, 537)	FieldSpec Pro FR spectroradiometer	Leaf N
Yao et al., 2014	Panicle initiation, stem elongation, and heading	NDVI	GreenSeeker sensor	Plant N
Moharana and Dutta, 2016	Booting, heading, and filling	R_705_/(R_717_ + R_491_)	FieldSpec Pro FR spectroradiometer	Leaf N
Qin et al., 2016	Jointing, heading, milking, ripening	D_738_/D_522_	HR-1024i	Leaf N

Most of previous studies focused on estimating N status in a dense canopy after the jointing stage and only a few evaluated the performance of LNC monitoring before canopy closure (Xue et al., 2004; Yu et al., 2013; Tian et al., 2014). The canopy spectra collected with field non-imaging spectrometers represent a mixture of reflectance signals from rice organs and water, soil or duckweed backgrounds in rice paddy fields (Gnyp et al., 2014; Sun et al., 2017). These environmental properties could negatively influence the spectroscopic estimation of LNC. In particular, the spectral properties of background materials dominate the overall canopy spectral signals during the early growth stages, which are critical periods for N fertilizer application and rice biomass formation (Nguyen and Lee, 2006; Tian et al., 2014). Furthermore, panicles gradually emerge from the sheath and are located in the upper-layer of rice canopies (Gnyp et al., 2014) at advanced reproductive stages. The coexistence of leaves and panicles in rice canopies makes the canopy reflectance signals more complicated and also creates uncertainties in the quantification of leaf N status from canopy reflectance spectra.

Recently, the use of ground-based or low-altitude UAV-based (unmanned aerial vehicle, UAV) multispectral and hyperspectral cameras has provided a promising avenue for implementing precision agriculture (Vigneau et al., 2011; Zhang and Kovacs, 2012) and crop phenotyping (Araus and Cairns, 2014). These cameras can provide a high spatial resolution with a range from a few millimeters to a few decimeters and high spectral resolution simultaneously. The spatial resolution (the smallest object that can be recognized from the imaging data) for different imaging sensors is determined by the size of IFOV (instantaneous field of view) and the altitude of the observation platform (Atkinson, 2017). This is directly related to the efficiency of data collection and processing, which can be improved as platform altitude increases and spatial resolution degrades (Jay et al., 2017). However, a high spatial heterogeneity might be observed within a coarser pixel size that can lead to a degree of uncertainty in the spectroscopic estimation of biochemical variables (Croft et al., 2013). Thus, the determination of optimal spatial resolution from sensitivity analyses is required for a specific application (Ming et al., 2011) such as the estimation of crop LNC to offer a good compromise between the estimation accuracy and the measurement efficiency of crop canopy images.

Ground-based imaging spectrometers can be used to collected images of crop canopies with an extremely high spatial resolution, which allowed us to do the sensitivity analysis using the strategy of upscaling for generating different spatial resolution imageries (Wu et al., 2009). With a simple setup of imaging spectrometers in the field, Jay et al. (2017) collected high resolution images of sugar beet canopies and successfully evaluate the sensitivity of chlorophyll content estimation to spatial resolution. However, their findings might be limited for monitoring rice N status because there were substantial differences in the canopy structure between sugar beet (with wider and more flat leaves) and paddy rice (with narrower and more tilt leaves).

For the estimation of crop LNC using ground-based hyperspectral systems (Li et al., 2014), previous studies were devoted to crops like wheat and sugar beet and focused only on observations of well-illuminated leaves (i.e., sunlit leaves) in the field (Vigneau et al., 2011; Jay et al., 2014). As a large proportion of rice leaves under natural light conditions in the field, shaded leaves were not considered in these experiments for the assessment of leaf or plant N status. Additionally, the hyperspectral images used in all of those studies (Vigneau et al., 2011; Jay et al., 2014) were obtained at either a single growth stage or over a narrow time window in the full growing season. It remains unclear how their models, based on multivariate regression methods (e.g., partial linear regression) and prediction approaches, would perform for other growth stages when canopy composition changes. Such concerns are evidenced by the fact that the relationships between the VIs of leaves and LNC were found to vary with the growth stages of rice (Xue et al., 2004; Yu et al., 2013). With the use of canopy reflectance spectra, Xue et al. (2004) and Yu et al. (2013) demonstrated that the VIs were well-correlated with LNC for individual stages but not so for pooled data from all stages.

In this regard, we focused on the LNC estimation in paddy rice canopies throughout the entire growing season using imaging spectroscopy data with various spatial resolutions ranging from millimeter- to centimeter-resolution. A total of 16 VIs commonly used for assessing N status were examined in terms of their relationships with LNC and their corresponding predictions of LNC for individual stages or stage-groups. Furthermore, we evaluated the feasibility of using multivariate methods for establishing regression models across all growth stages. The specific research objectives were: (1) to compare the LNC~VI relationships and validate the predictive performance for individual growth stages or stage-groups at each spatial resolution; (2) to evaluate the sensitivity of global LNC estimation to spatial resolution using multivariate regression methods across all growth stages; (3) to determine the optimal spatial resolution for the LNC estimation in rice canopies.

## Materials and methods

### Experimental design

Two rice (*Oryza sativa* L.) experiments were designed, encompassing a combination of treatments of rice cultivars, planting density, and N rate. These two experiments were respectively conducted in 2014 and 2015 from July to September at the experimental station of National Engineering and Technology Center for Information Agriculture (NETCIA), Rugao, Jiangsu, China (120°19′ E, 32°14′ N) with the same treatments for each year. The predominant soil texture was loam and the organic carbon concentration in soil was 12.95 g·kg^−1^. The annual average temperature was 14.6°C and annual average precipitation was 1055.5 mm, respectively.

Four N fertilization rates [0 (N0), 100 (N1), 200 (N2), and 300 (N3) kg N ha^−1^] were applied in the form of urea, with 40% at preplanting, 10% at tillering, 30% at jointing and 20% at booting. In particular, there were two planting densities (0.30 m by 0.15 m and 0.50 m by 0.15 m) for N1 and N2 rates and one planting density (0.30 m by 0.15 m) for N0 and N3 rates. Each treatment had three replicates which were arranged in a randomized block design. For all plots, 135 kg P_2_O_5_ ha^−1^ (as phosphate fertilizer) and 190 kg K_2_O ha^−1^ (as potassium fertilizer) were applied before transplanting. The two rice cultivars were Japonica rice of erect plant type, Wuyunjing 24 (V1), and Indica rice of spread type, Y liangyou 1 (V2). Each plot size was 5 m by 6 m and a total of 36 plots (12 cultivation conditions with three replications) were grown in each experiment.

### Acquisition and preprocessing of hyperspectral imagery

#### Hyperspectral image data acquisition

**Figure 1:**
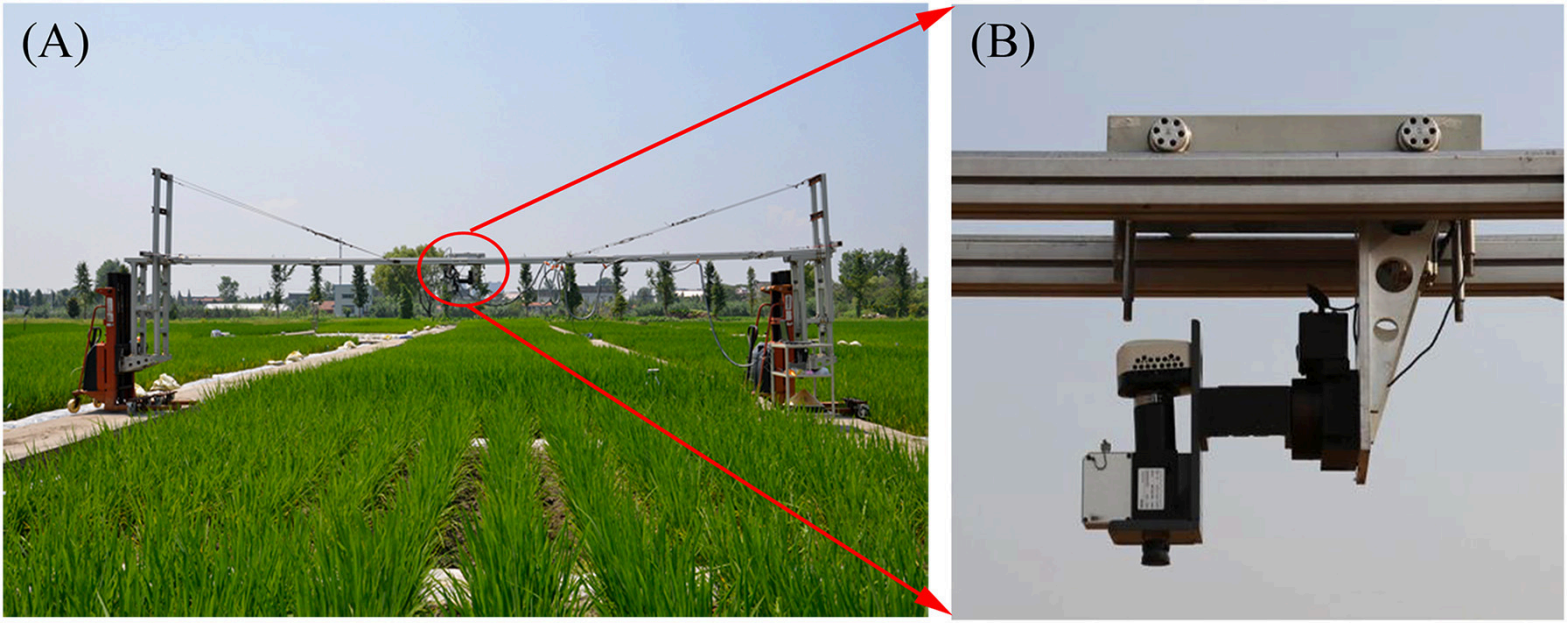
**(A)** Experimental setup of the near-ground hyperspectral imaging system in the paddy field and **(B)** onset of the hyperspectral camera in the system.

**Figure 2:**
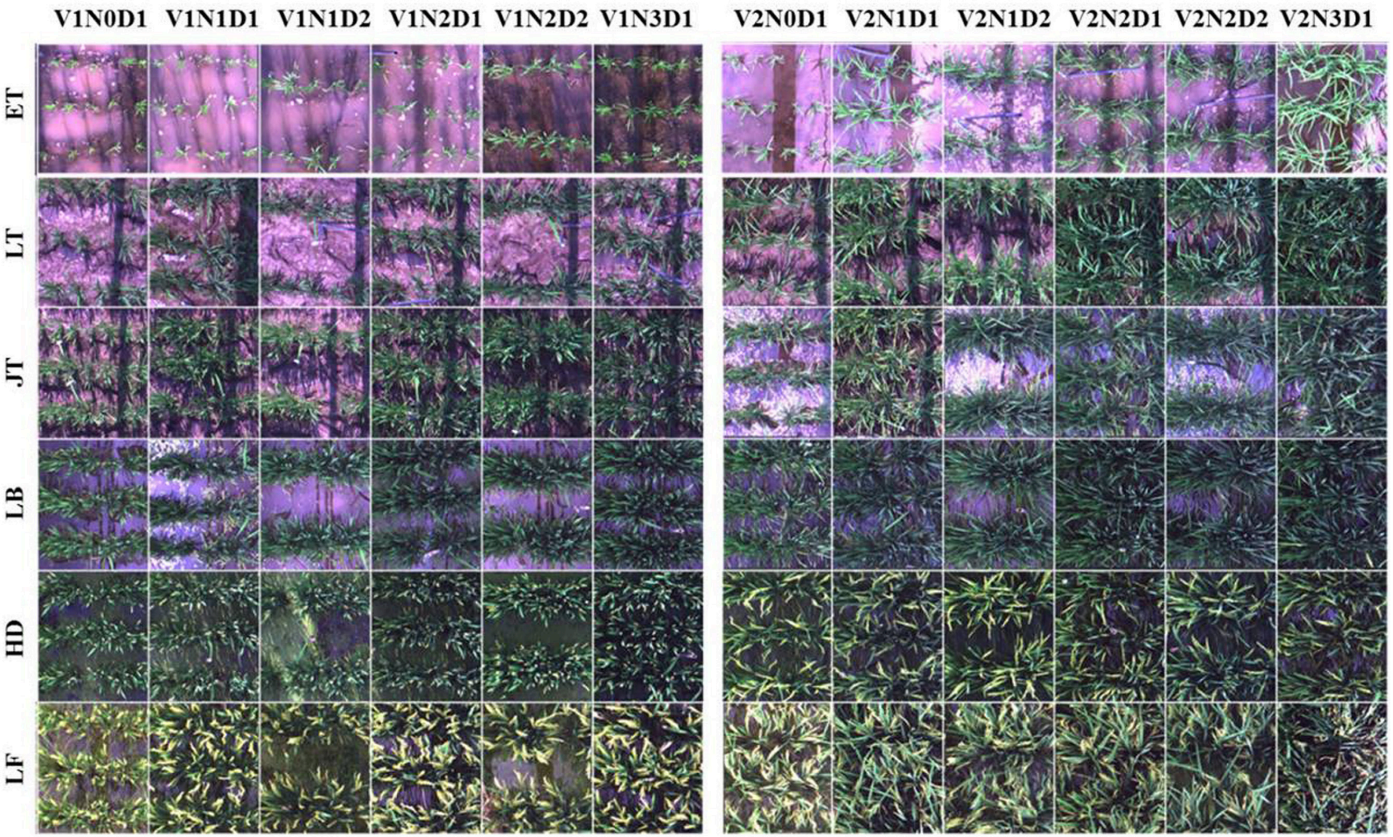
Example true color images cropped from hyperspectral scenes acquired throughout the growing season in 2014. A total of 12 plots (one replication) for each growth stage were shown here. V1: Wuyunjing 24 (Japonica rice); V2: Y liangyou 1 (Indica rice). N0-N3: four N fertilization rates (0, 100, 200, and 300 kg·N·ha^−1^). D1-D2: two row spacings (30 and 50 cm). ET, early tillering stage; LT, late tillering stage; JT, jointing stage; LB, late booting stage; HD, heading stage; LF, late filling stage.

All hyperspectral images were acquired by a pushbroom scanning sensor (ImSpector V10E-PS, SpecIm, Finland) mounted on a platform about 1.2 m above the rice canopies (Figure 1). Our platform could be lifted up to a maximum height of 3 m above the ground and we could ensure fields of view in the same size at the top of the canopy throughout the entire growing season. The spectral range of this sensor was from 360 to 1025 nm divided into 520 bands with a spectral resolution of 2.8 nm. The spatial dimension of image data was acquired by the movement of a linear actuator. The spatial resolution at near-nadir position (42.8°Field of view) was about 1.3 mm and the swath width was about 0.9 m. The exposure time of this sensor was fixed manually to adapt to brightness variation between scans under natural light conditions, with ~0.2 ms for sunny days. This hyperspectral imaging system completed a scene by scanning rice canopies across the row orientation (5 m wide) and generated a total of 432 images for the two experiments. In particular, 36 images were collected for each growth stage (Figure 2). The summary of image acquisition dates is shown in Table 2.

**Table 2:** Summary of image acquisition dates (also denoted as days after transplanting: DAT) for the rice experiment.

**Years**	**Early tillering**	**Late tillering**	**Jointing**	**Early booting**	**Late booting**	**Heading**	**Early filling**	**Late filling**
2014	8 Jul. (22)	20 Jul. (34)	4 Aug. (49)	/	20 Aug. (65)	3 Sept. (78)	/	20 Sept. (96)
2015	9 Jul. (24)	21 Jul. (36)	31 Jul. (46)	14 Aug. (60)	25 Aug. (71)	/	9 Sept. (86)	/

#### Data preprocessing

The image preprocessing procedures including subtraction of sensor electronic noise (dark current) and radiometric correction were implemented within the specVIEW software (Specim, Oulu, Finland). The final relative reflectance values were converted from the original digital number (DN) values (i.e., pixel brightness values) using the calibration equation as follows (Zhou et al., 2017):

(1) $$\begin{array}{r} Ref_{\text{target}}=\frac{DN_{\text{target}}-DN_{\text{noise}}}{DN_{\text{panel}}-DN_{\text{noise}}}\times Ref_{\text{panel}} \end{array}$$

where, *DN*_target_, *DN*_noise_, and *DN*_panel_ is the DN value of target, electronic noise (dark current) and 99% reflective white reference panel, respectively. *Ref*_target_ and *Ref*_panel_ is the reflectance value of target and reference panel, respectively. A barium sulfate (BaSO_4_) panel was placed on the tripod as the white reference panel Herrmann et al., 2013. The relative reflectance data were smoothed using the Minimum Noise Fraction (MNF) transform procedure in the ENVI 4.8 (EXELIS, Boulder, CO, USA) software environment. The spectral data in the 400–900 nm range were retained because of strong noise in other spectral regions even after smoothing.

#### Image spatial subsampling

**Figure 3:**
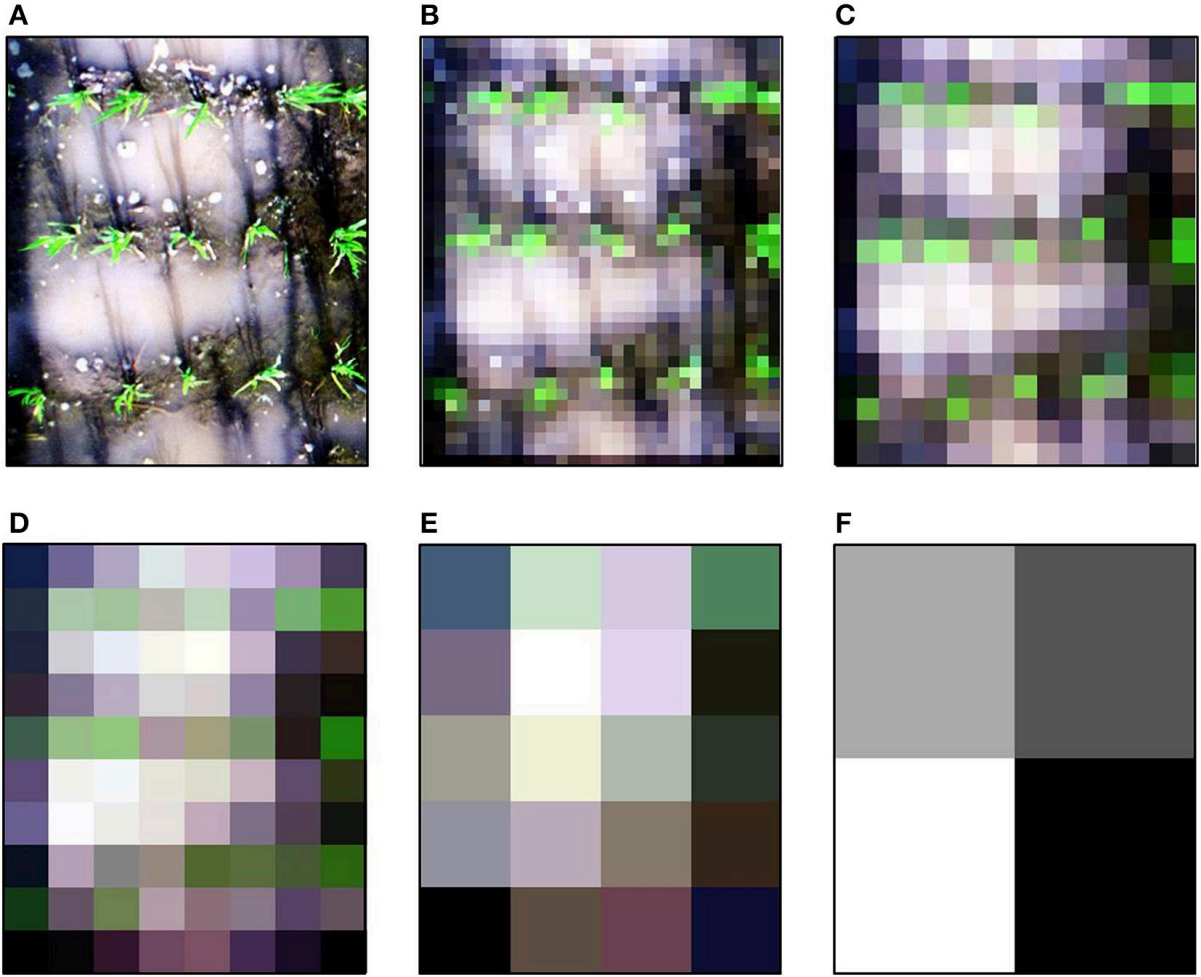
Example true color images with gradual degradation of spatial resolution acquired on 8 July 2014. Illustration for **(A)** 1.3 mm, **(B)** 14 mm, **(C)** 28 mm, **(D)** 56 mm, **(E)** 113 mm and **(F)** 225 mm.

To avoid the bi-directional reflectance distribution reflectance (BRDF) effects on the left and right edges of images caused by the impact of wide viewing geometry, we cropped original images and only used the middle half of the original images (i.e., the swath width was about 45 cm). The performance of LNC estimation was evaluated over various spatial resolutions. Given the swath width was 450 mm, we selected the spatial resolutions by dividing 450 mm by a factor of two iteratively. The original spatial resolutions of 1.3 mm was degraded to 2, 4, 7, 14, 28, 56, 113, 225, 450 mm by aggregating over cells of N × N pixels as suggested in Jay et al. (2017). An example image at different spatial resolutions is illustrated in Figure 3.

#### Discrimination of non-vegetation background and vegetation

To investigate the relationships between LNC and VIs derived from pure leaf pixels across the whole image at 1.3 mm spatial resolutions, we firstly identified vegetation pixels applying a threshold of the enhanced vegetation index (EVI) (Pinto et al., 2016; Zhou et al., 2017) (EVI > 0.45). The EVI is an optimized vegetation index with improved vegetation monitoring through minimizing the effects of background influences and atmosphere influences (Liu and Huete, 1995). Its discrimination capacity for separating non-vegetation background and vegetation pixels has been proved in previous studies (Pinto et al., 2016; Zhou et al., 2017). Afterwards, we constructed the classification decision tree developed in Zhou et al. (2017) by applying photochemical reflectance index (PRI) (Gamon et al., 1992) and transformed chlorophyll absorption reflectance index (TCARI) (Haboudane et al., 2002) thresholds at two sequential steps for discriminating all the pixels of sunlit and shaded canopy leaves and panicles in the images. The PRI was originally developed to track the current de-epoxidation state of the xanthophyll pigment and used as a proxy of light use efficiency (LUE) in many studies (Gamon et al., 1992; Cheng et al., 2012; Damm et al., 2015). The PRI values for panicles were substantially lower than those for leaves because of the substantially less efficient photosynthesis and systematically lower light use efficiency for panicles (Zhou et al., 2017). The TCARI had been widely used as a proxy of vegetation chlorophyll content (Haboudane et al., 2002). The differences in the strength of apparent chlorophyll absorption led to clear separations of sunlit and shaded counterparts throughout the whole growing season (Kokaly et al., 2003; Castro and Sanchez-Azofeifa, 2008; Zhou et al., 2017). Specifically, we extracted all the leaf pixels with PRI > −0.058 and all panicle pixels with PRI ≤ −0.058. Then, the pixels of sunlit leaves and shaded leaves were identified with TCARI values greater and less than 0.172, respectively; the sunlit and shaded panicles were identified with a TCARI value of greater and lower than 0.241, respectively. More detailed information regarding the discrimination of different components within rice canopies could be found in Zhou et al. (2017).

**Figure 4:**
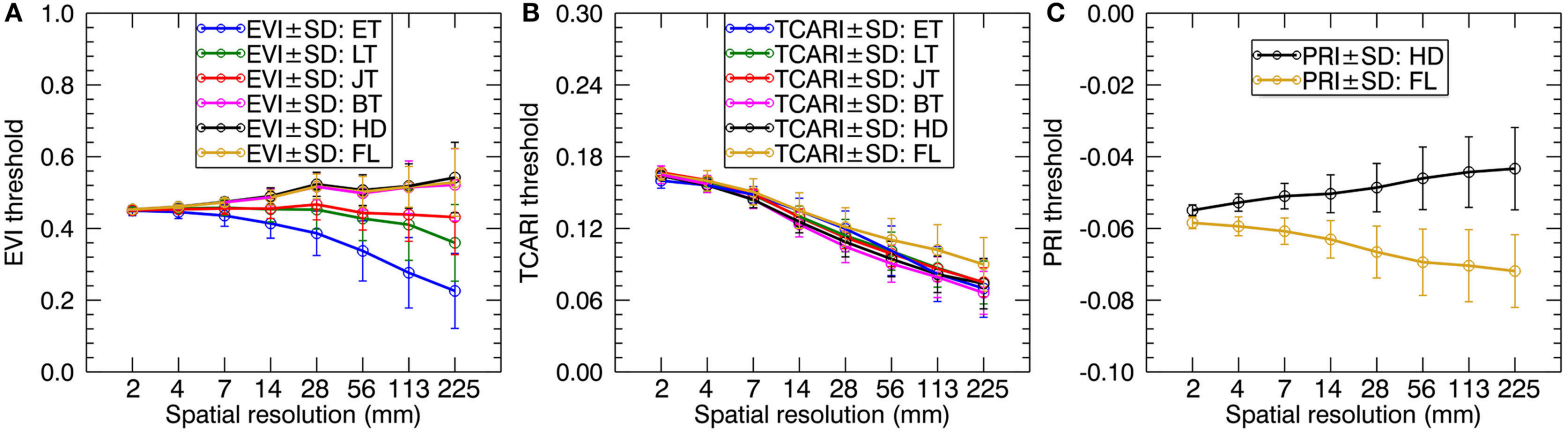
The profiles of EVI **(A)**, TCARI **(B)**, and PRI **(C)** threshold values adjusted for coarser resolutions to keep the similar green fraction. ET, early tillering stage; LT, late tillering stage; JT, jointing stage; BT, booting stage; HD, heading stage; FL, filling stage.

We calculated the green fraction (the fraction of green vegetation pixels observed by the sensor) from the original images with 1.3 mm spatial resolution for which the fraction of mixed pixels was negligible. Then, we followed the strategy in Jay et al. (2017) to adjust the threshold values of EVI, TCARI, and PRI for individual resolutions to keep the green fraction close to that calculated at the 1.3 mm spatial resolution. As shown in Figure 4, the EVI threshold values decreased as the spatial resolution degraded for the early tillering stage and the late tillering stage but slightly increased for the reproductive stages. In contrast, TCARI threshold values exhibited a tendency to decline from 2 mm resolution to 225 mm resolution for each stage. The PRI threshold values increased with the degradation of spatial resolution for the heading stage but decreased for the filling stage. Given only one pixel left when degrading the original cropped image to 450 mm spatial resolution, we did not provide the separate thresholds for this resolution. Additionally, the average spectra of all-leaf pixels, sunlit-, and shaded-leaf pixels for 450 mm spatial resolution were assigned as the average spectra of whole-image pixels [vegetation pixels and background pixels) across the original cropped image (1.3 mm spatial resolution)].

### Calculation of VIs

VIs are designed with band combinations to amplify their sensitivity toward particular biochemical or biophysical parameters and while also minimize the possible confounding effects (Malenovský et al., 2015). We included indices from the literature, specifically the evaluation of N status of crops based on leaf and canopy level reflectance. We selected three types of published VIs, including the ratio indices, the normalized difference indices, and the combined vegetation indices to estimate LNC as listed in Table 3. These three types of indices have been commonly used for monitoring vegetation nitrogen status. In this study, reflectance spectra were firstly averaged over all pixels for different types of leaf pixels (i.e., all-leaf, sunlit-leaf, and shaded-leaf) at each spatial resolution and the VIs were then calculated. The band selection for each index was deliberately implemented as suggested in Yu et al. (2013) by involving different spectral ranges for blue, red, green, red-edge, and NIR.

**Table 3:** Published VIs related to N status of crops used in this study.

**Index**	**Equation**	**References**
		
**SIMPLE RATIO INDICES**		
SR[800, 675]	R_800_/R_675_	Jordan, 1969
SR[810, 560]	R_810_/R_560_	Xue et al., 2004
SR[750, 550]	R_750_/R_550_	Kim et al., 1994
SR[750, 710]	R_750_/R_710_	Zarco-Tejada et al., 2001
CI_Red−edge_	R_800_/R_720_ −1	Gitelson et al., 2003
**NORMALIZED DIFFERENCE INDICES**		
NDVI	(R_800_-R_670_)/(R_800_+R_670_)	Rouse et al., 1974
GNDVI	(R_750_-R_550_)/(R_750_+R_550_)	Gitelson et al., 1996
ND_705_	(R_750_-R_705_)/(R_750_+R_705_)	Gitelson and Merzlyak, 1994
mND_705_	(R_750_-R_705_)/(R_750_+R_705_-2^*^R_445_)	Sims and Gamon, 2002
mSR_705_	(R_750_-R_445_)/(R_705_-R_445_)	Sims and Gamon, 2002
MTCI	(R_750_-R_710_)/(R_710_-R_680_)	Dash and Curran, 2004
PRI	(R_531_-R_570_)/(R_531_+R_570_)	Gamon et al., 1992
**COMBINED VEGETATION INDICES**		
TCARI	3^*^[(R_700_-R_670_)-0.2^*^(R_700_-R_550_)(R_700_/R_670_)]	Haboudane et al., 2002
OSAVI	(1+0.16)(R_800_-R_670_)/(R_800_+R_670_+0.16)	Rondeaux et al., 1996
TCARI/OSAVI	TCARI/OSAVI	Haboudane et al., 2002
DCNI	(R_720_-R_700_)/(R_700_-R_670_)/(R_720_-R_670_+0.03)	Chen et al., 2010

Ratio indices represent the simplest type. Ratio indices are calculated as the ratio of reflectance in two wavebands: the first band is sensitive to pigment absorption features and leaf scattering; the second band is only sensitive to leaf scattering and used as a reference band to minimize leaf scattering effects (Blackburn, 2007; Jay et al., 2017). For example, CI_Red−edge_ (Gitelson et al., 2003) is a commonly used ratio index for estimating chlorophyll content in crops. CI_Red−edge_ combines a red-edge band that is well-correlated to chlorophyll content (Horler et al., 1983) and a reference band located in the near-infrared domain.

The normalized difference indices are calculated with a few wavebands (commonly two or three wavebands): the first waveband is sensitive to a target parameter (e.g., chlorophyll content); other bands are used to minimize the influence of leaf scattering, leaf surface (specular) reflection, soil background, or atmosphere for enhancing the relationships with the target parameter. For example, mND_705_ combines a red-edge band (705 nm) that is sensitive to chlorophyll content (Lamb et al., 2002) with a near-infrared shoulder band (750 nm) and a blue band (445 nm) to minimize the influences of leaf scattering and specular reflection, respectively.

The combined vegetation indices are built based on a few spectral indices: the first index is sensitive to a target parameter; other indices are used to minimize the influence of soil background or canopy structure for enhancing the relationships with the target parameter. For example, TCARI/OSAVI (Haboudane et al., 2002) has been commonly used to estimate crop chlorophyll content. Specifically, the transformed chlorophyll absorption ratio index (TCARI) (Haboudane et al., 2002) and the optimized soil-adjusted vegetation index (OSAVI) (Rondeaux et al., 1996) are related to leaf chlorophyll content and leaf area index, respectively. When the TCARI combined with the OSAVI, the relationships with leaf chlorophyll content can be strengthened by reducing the influence of soil background and canopy structure.

### Multivariate methods

Using multivariable methods is helpful for taking advantage of hyperspectral data with large numbers of wavebands at fine spectral resolution (Inoue et al., 2012).

#### Partial least squares regression (PLSR)

Partial Least Squares Regression (Martens and Næs, 1989; Wold et al., 2001) is one of the reliable analytical tools for multivariable data analysis and have been widely used in the assessment of crop nitrogen status (Vigneau et al., 2011; Inoue et al., 2012; Ecarnot et al., 2013; Yu et al., 2014). It possesses an advantage to avoid high multi-collinearity among variables, which is the inherent issue in stepwise multiple linear regression (Inoue et al., 2012). When compared with multiple stepwise regression or principal component, PLSR generally exhibits better predictive performance (Ye et al., 2008; Ecarnot et al., 2013; Yu et al., 2014). Specifically, PLSR models are built based on latent variables instead of real variables (Yu et al., 2014).

#### Gaussian process regression (GPR)

GPR is a non-parametric method that learns the relationship between the input variables (e.g., reflectance) and output parameters (e.g., LNC) by fitting a flexible probabilistic (Bayesian) model directly in function space, with no intermediate model or model parameters (Verrelst et al., 2012, 2013). Over the last decade, GPR has emerged as an effective machine learning approach to retrieving biophysical parameters (Verrelst et al., 2012, 2013, 2016). In particular, GPR has been used for mapping leaf area index and fractional vegetation cover (Verrelst et al., 2012) and quantifying vegetation traits such as leaf chlorophyll content (Verrelst et al., 2012, 2013) and canopy water content (Verrelst et al., 2016).

GPR has alleviated some shortcomings of similar machine learning methods, while generally achieving good predictive performances and stabilities. For example, training GPR is far simpler than neural networks or support vector machines by using very flexible kernels with several free parameters. Furthermore, GPR provides a ranking of features (e.g., wavelengths) and samples (e.g., reflectance spectra) and thus partly overcoming the blackbox problems encountered in non-parametric regression methods (Verrelst et al., 2012).

### LNC measurements

After each measurement of canopy hyperspectral images, three clusters of plants at the center of the spectral sampling area from each plot were selected randomly and destructively sampled for the determination of leaf weight and LNC. For each sample, all green leaves were separated from their stems, and oven-dried for 30 min at 105°C, and then for about 24 h at 80°C till constant weight. Dried leaf samples were ground and then stored in plastic bags prior to chemical analysis. LNC (%) was determined with micro-Keldjahl analysis method (Tian et al., 2014).

### Calibration and validation of predictive models

The predictive models were divided into two types: stage-specific and stage-non-specific (i.e., global models that are suitable for the full season). Specifically, the stage-specific models for individual growth stages before booting or stage-groups after booting were constructed using simple linear or non-linear regression analysis between LNC and VIs derived from the average spectra of all the leaf pixels, sunlit, or shaded leaf pixels for individual spatial resolution datasets.

The global models were constructed using two multivariable methods: PLSR (Martens and Næs, 1989; Wold et al., 2001) and GPR (Rasmussen and Williams, 2006) based on the samples across all growth stages. Specifically, we calibrated models between LNC and reflectance spectra (400–900 nm) or continuum-removed reflectance spectra (550–750 nm) (Kokaly et al., 2003) using PLSR and GPR, respectively. Additionally, we selected the optimal number of latent variables for calibrating the PLSR model by leave-one-out cross-validation on the calibration set.

Two replications for each treatment were used for calibrating predictive models (i.e., 2/3 of the samples used as the calibration samples) and one replication (i.e., 1/3 of the samples used as the validation samples) were used for validating the models. With regard to the stage-specific models, there were 24 calibration samples and 12 validation samples for individual growth stages before booting but 144 calibration samples and 72 validation samples for the stage-group after booting (i.e., reproductive stages). For the global models, there were 288 calibration samples and 144 validation samples through the whole growing season. The performance of models was assessed using the predictive coefficient of determination (R^2^), root mean square error (RMSE) between the measured and predicted LNC values.

## Results

### Seasonal variation in LNC and spectral properties of all-leaf pixels and whole-image pixels

**Figure 5:**
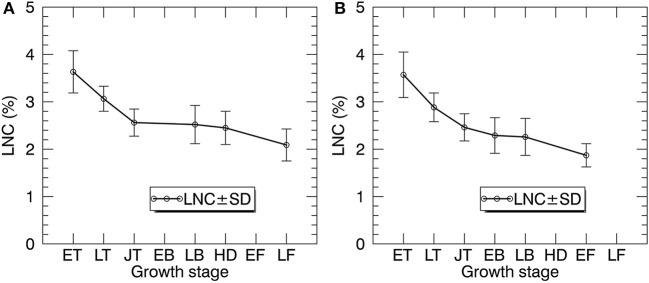
The temporal profiles of LNC in paddy rice over the whole season in 2014 **(A)** and 2015 **(B)**. ET, early tillering stage; LT, late tillering stage; JT, jointing stage; EB, early booting stage; LB, late booting stage; HD, heading stage; EF, Early filling stage; LF, Late filling stage.

Figure 5 shows the statistics of LNC measurements for individual growth stages in 2014 and 2015. Generally, the LNC values for 2014 and 2015 decreased from 4.29 to 1.36% with the development of growth stages. For both years, LNC changed dramatically from early tillering to jointing stages.

**Figure 6:**
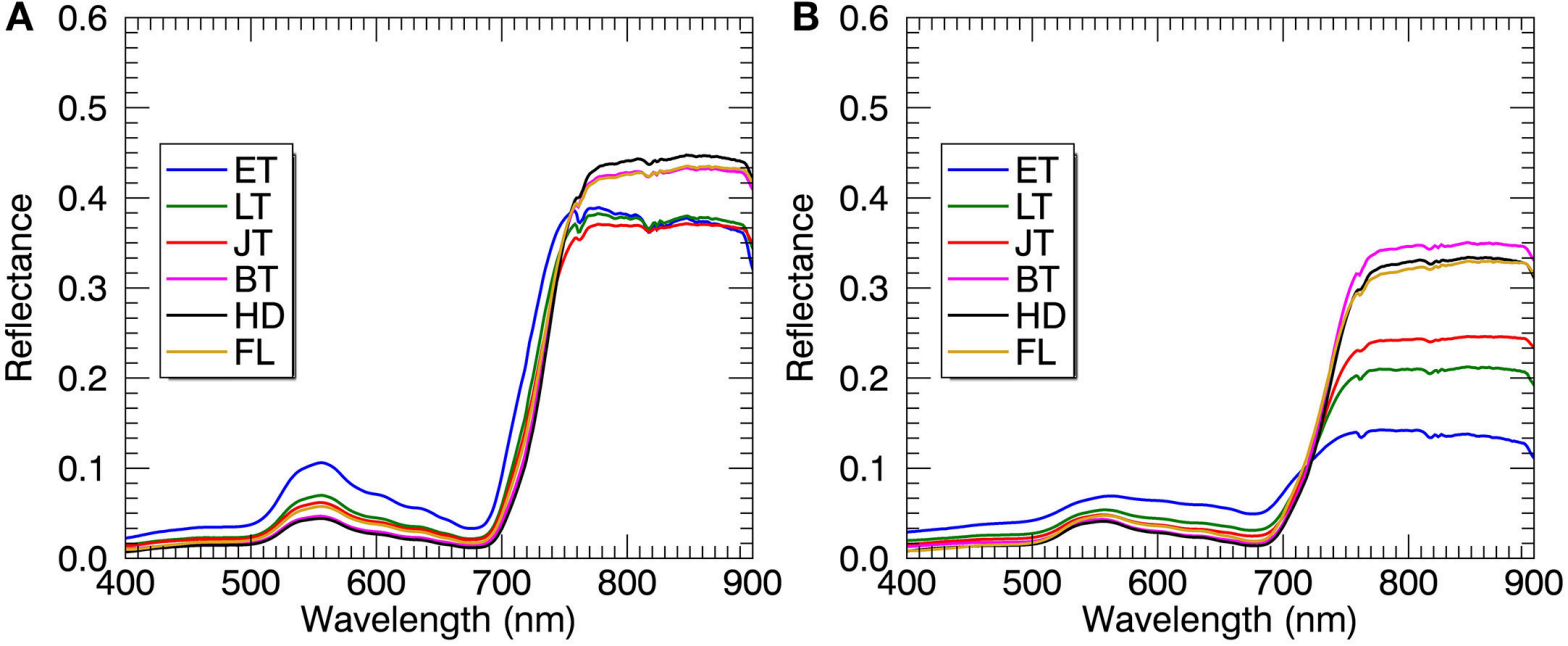
Mean reflectance spectra derived from all-leaf pixels **(A)** and whole-image pixels **(B)** at individual growth stages. Plots represent the combined data from 2014 and 2015. ET, early tillering stage; LT, late tillering stage; JT, jointing stage; BT, booting stage (including early and later booting stages); HD, heading stage; FL, filling stage (including early and later filling stages).

Regardless of spectral datasets for all-leaf pixels and whole-image pixels, the average reflectance of all plots in the visible region decreased from the early tillering stage to the booting or heading stage and then raised until the filling stage (Figure 6). However, the reflectance in the NIR region showed an opposite tendency as compared to the visible region. With regard to the comparison between the reflectance spectra of all-leaf pixels and whole-image pixels for individual stages, the NIR reflectance spectra averaged over all the leaf pixels exhibited higher amplitudes than those averaged over all canopies, especially during the early growth stages.

### Relationships of rice LNC with VIs derived from all-leaf pixels and whole-image pixels for various stages

Table 4 shows a summary of squared Spearman's correlation coefficients for all VIs relating to LNC by growth stage. From the early tillering stage to the jointing stage, most of the correlations (*p* < 0.05) for the VIs derived from all-leaf pixels exhibited higher *R*^2^-values than those derived from whole-image pixels. This contrast generally became less significant with the development of growth stages but was still apparent at the group of reproductive stages. Among the VIs examined, the ones employing red-edge bands (i.e., SR [750, 710], ND_705_, mSR_705_, MTCI, mND_705_, CI_Red−edge_) displayed higher *R*^2^-values than others. CI_Red−edge_, MTCI and TCARI/OSAVI represented the best performing VIs for the three groups, respectively. With regard to the performance of these three representative VIs, TCARI/OSAVI generally exhibited higher ρ^2^ than CI_Red−edge_ and MTCI at the late tillering stage but lower ρ^2^ at the reproductive stages. In contrast, these three VIs exhibited similar ρ^2^-values for remaining two stages (early tillering and jointing).

**Table 4:** Squared correlation coefficients (ρ2) from Spearman's correlation for the relationships between LNC and VIs derived from all-leaf pixels or whole-image pixels (leaf + background pixels) of the original images at 1.3 mm spatial resolution for individual growth stages in the vegetative period and the group of reproductive stages.

**VIs**	**Early tillering**		**Late tillering**		**Jointing**		**Reproductive**	
	**Leaf pixels**	**Leaf +background pixels**	**Leaf pixels**	**Leaf +background pixels**	**Leaf pixels**	**Leaf +background pixels**	**Leaf pixels**	**Leaf +background pixels**
**SIMPLE RATIO**								
SR[800, 675]	0.27^**^	0.27^**^	0.43^**^	0.23^**^	0.42^**^	0.37^**^	0.43^**^	0.28^**^
SR[810, 560]	0.49^**^	0.29^**^	0.46^**^	0.28^**^	0.58^**^	0.47^**^	0.64^**^	0.46^**^
SR[750, 550]	0.49^**^	0.26^**^	0.45^**^	0.27^**^	0.55^**^	0.44^**^	0.59^**^	0.40^**^
SR[750, 710]	0.69^**^	0.35^**^	0.59^**^	0.34^**^	0.66^**^	0.52^**^	0.67^**^	0.53^**^
CI_Red−edge_	0.71^**^	0.39^**^	0.61^**^	0.40^**^	0.67^**^	0.58^**^	0.70^**^	0.61^**^
**NORMALIZED DIFFERENCE INDEX**								
NDVI	0.27^**^	0.27^**^	0.41^**^	0.23^**^	0.42^**^	0.37^**^	0.47^**^	0.31^**^
GNDVI	0.49^**^	0.26^**^	0.45^**^	0.27^**^	0.55^**^	0.44^**^	0.59^**^	0.40^**^
ND_705_	0.69^**^	0.35^**^	0.60^**^	0.31^**^	0.64^**^	0.49^**^	0.65^**^	0.50^**^
mND_705_	0.62^**^	0.39^**^	0.69^**^	0.36^**^	0.69^**^	0.57^**^	0.71^**^	0.59^**^
mSR_705_	0.62^**^	0.39^**^	0.69^**^	0.36^**^	0.69^**^	0.57^**^	0.71^**^	0.59^**^
MTCI	0.70^**^	0.41^**^	0.64^**^	0.52^**^	0.69^**^	0.63^**^	0.71^**^	0.63^**^
PRI	0.05	0.12^*^	0.01	0.08	0.08	0.29^**^	0.51^**^	0.36^**^
**COMBINED DIFFERENCE INDEX**								
TCARI	0.42^**^	0.06	0.64^**^	0.01	0.75^**^	0.06	0.66^**^	0.33^**^
OSAVI	0.15^**^	0.25^**^	0.07	0.18^**^	0.37^**^	0.39^**^	0.36^**^	0.22^**^
TCARI/OSAVI	0.64^**^	0.47^**^	0.76^**^	0.67^**^	0.72^**^	0.60^**^	0.69^**^	0.52^**^
DCNI	0.48^**^	0.28^**^	0.62^**^	0.49^**^	0.75^**^	0.56^**^	0.63^**^	0.48^**^

* P < 0.01;

** *P < 0.001. The highest correlations in each column are highlighted in bold*.

**Figure 7:**
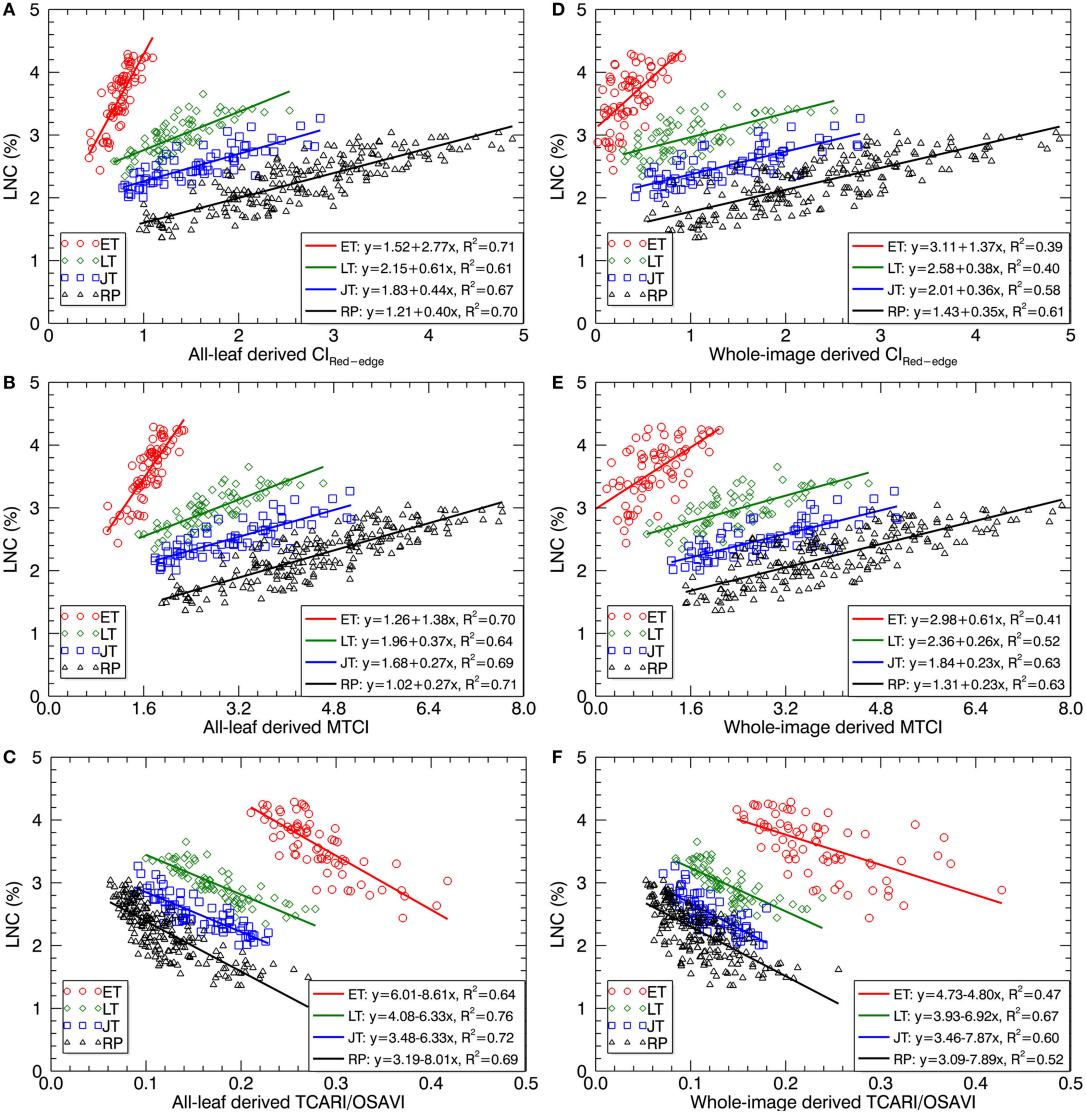
Best-fit linear relationships of LNC with three representative VIs derived from all-leaf pixels and whole-image pixels: **(A,D)** CI_Red−edge_, **(B,E)** MTCI and **(C,F)** TCARI/OSAVI across the original cropped images at 1.3 mm spatial resolution. ET, early tillering stage; LT, late tillering stage; JT, jointing stage; RP, reproductive stages (stages after booting).

Figure 7 shows the scatter plots of LNC~VI models with three representative VIs derived from all-leaf pixels and whole-image pixels for different growth stages. Within both all-leaf pixels and whole-image pixels represented by the three VIs, the LNC~VI models were different among the first three stages and another single model could fit for the remainder (reproductive phase) of growth stages. From early tillering to reproductive stages, the LNC decreased substantially but the VIs did not follow the decrease, which led to the presence of four clusters in each of the scatter plots. Specifically, the scatter plots of the VIs derived from all-leaf pixels were more concentrated than those of the VIs derived from whole-image pixels for any of the stages before booting, with the most significant discrepancy being for the early tillering stage. While the LNC~VI models for the stages of late tillering and jointing exhibited similar slopes to those for the reproductive stages, the models for the early tillering stage differed from all of them in slope and intercept. These model differences between stages precluded the data from being fitted with a global model for the whole season. A multi-stage model was possible only for data from the post-booting stages as shown in Figure 7 and Table 4. For the correlations with LNC in the pooled data over reproductive stages, most indices derived from all-leaf pixels exhibited marginal differences in *R*^2^ as compared to those derived from whole-image pixels except TCARI.

### Sensitivity of the stage-specific LNC~VI relationships to spatial resolution

**Figure 8:**
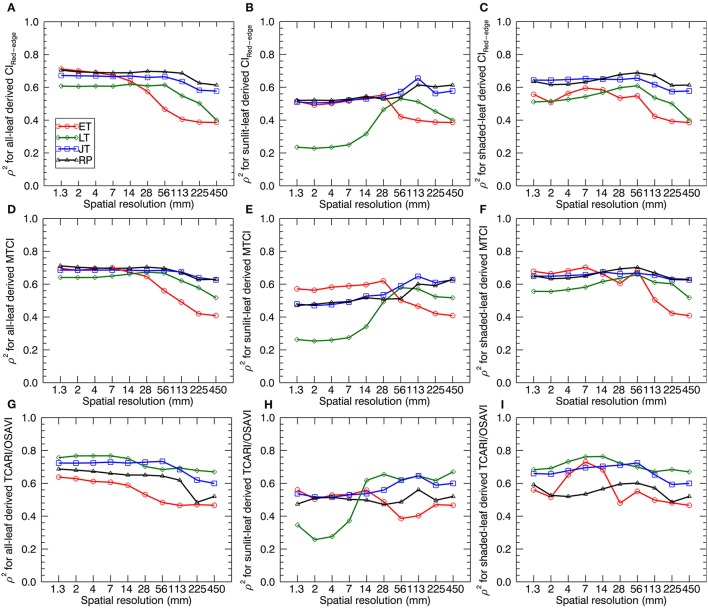
The squared Spearman's correlation (ρ^2^) between three representative VIs and LNC as a function of spatial resolution. VIs are derived from the average reflectance spectra of all-leaf pixels, sunlit- and shaded-leaf pixels across the whole image for different growth stages. The VIs for all-leaf pixels, sunlit- and shaded-leaf pixels at 450 mm spatial resolution were all calculated from the average reflectance spectra of whole-image pixels. ET, early tillering stage; LT, late tillering stage; JT, jointing stage; RP, reproductive stages (stages after booting). **(A–C)** CI_Red−edge_ derived from all-, sunlit- and shaded-leaf pixels. **(D–F)** MTCI derived from all-, sunlit- and shaded-leaf pixels. **(G–I)** TCARI/OSAVI derived from all-, sunlit- and shaded-leaf pixels.

Figure 8 shows the sensitivity (in terms of ρ^2^) of LNC~VI relationships (derived from all-leaf pixels, sunlit-, and shaded-leaf pixels) to spatial resolution for different growth stages. Generally, the correlations of VIs with LNC decreased with the degradation of spatial resolutions at a specific stage (or stage-group). Specifically, most ρ^2^-values for the early tillering stage remained nearly stable from 1.33 to 14 mm spatial resolutions, except for a maximum (ρ^2^ = 0.73 as the highest value for the early tillering stage) being observed at 7 mm spatial resolution for TCARI/OSAVI derived from shaded-leaf pixels. In contrast, most ρ^2^-values decreased substantially from 14 to 450 mm spatial resolution regardless of leaf pixel types. The changing patterns of ρ^2^ for the late tillering, jointing, and reproductive stages were generally similar to that of the early tillering stage except the decline starting from 56 mm spatial resolution for VIs derived from all-leaf pixels and shaded-leaf pixels but from 113 mm spatial resolution for VIs derived from sunlit-leaf pixels.

Comparing three types of leaf pixels, most VIs of sunlit leaves displayed weaker relationships with LNC than those of all leaves and shaded leaves. In particular, VIs of sunlit leaves exhibited much lower correlations with LNC at the late tillering stage for 1.3 ~ 14 mm spatial resolution (ρ^2^ = 0.23~0.62). In contrast, more stable values of ρ^2^ were observed for considering all-leaf pixels over different spatial resolutions. Especially, MTCI derived from all-leaf pixels exhibited closet ρ^2^ between different spatial resolutions at specific stages.

As shown in Table 5, the best performing VI for all the individual stages before booting was TCARI/OSAVI. In particular, the best models were observed at a finer resolution for the early tillering stage (shade-leaf pixels at 7 mm spatial resolution) and the late tillering stage (all-leaf pixels at 4 mm spatial resolution) but at a coarser resolution for the jointing stage (all-leaf pixels at 56 mm spatial resolution). For the reproductive stages, MTCI derived from all-leaf pixels at 28 mm spatial resolution performed better than other situations.

**Table 5:** The prediction accuracies for the best-fit linear relationships between LNC and a specific VI from three representative VIs at a specific spatial resolution for the early tillering stage, the late tillering stage, the jointing stage, and reproductive stages.

**Growth stages**	**Best VIs**	***R*^2^**	**RMSE (%)**
Early tillering	TCARI/OSAVI (Shaded-leaf pixels, 7 mm)	0.65	0.25
Late tillering	TCARI/OSAVI (All-leaf pixels, 4 mm)	0.66	0.18
Jointing	TCARI/OSAVI (All-leaf pixels, 56 mm)	0.67	0.18
Reproductive stages	MTCI (All-leaf pixels, 28 mm)	0.72	0.23

### Sensitivity of global LNC predictions to spatial resolution

When applying the global models (i.e., stage-non-specific models for all stages) to the validation dataset, the prediction accuracies generally decreased with the degradation of spatial resolution. The *R*^2^ for all-leaf pixels slightly changed from 1.3 to 28 mm (e.g., *R*^2^ = 0.69~0.72 for GPR using reflectance spectra of all-leaf pixels) and then gradually decreased. Specifically, all-leaf pixels exhibited more stable predictive accuracies over different spatial resolutions (*R*^2^ = 0.55~0.73 for GPR; *R*^2^ = 0.50~0.63 for PLSR) than sunlit-leaf pixels (*R*^2^ = 0.43~0.75 for GPR; *R*^2^ = 0.45~0.63 for PLSR) and shaded-leaf pixels (*R*^2^ = 0.53~0.68 for GPR; *R*^2^ = 0.42~0.60 for PLSR). In contrast, the accuracies for the sunlit-leaf pixels dramatically declined with the degradation of spatial resolutions after 7 mm spatial resolution. In particular, sunlit-leaf pixels produced the best prediction of LNC at 1.3 ~ 4 mm spatial resolution (*R*^2^ > 0.74, RMSE < 0.33 %) while performing GPR regardless of using reflectance spectra and continuum-removed reflectance spectra.

**Figure 9:**
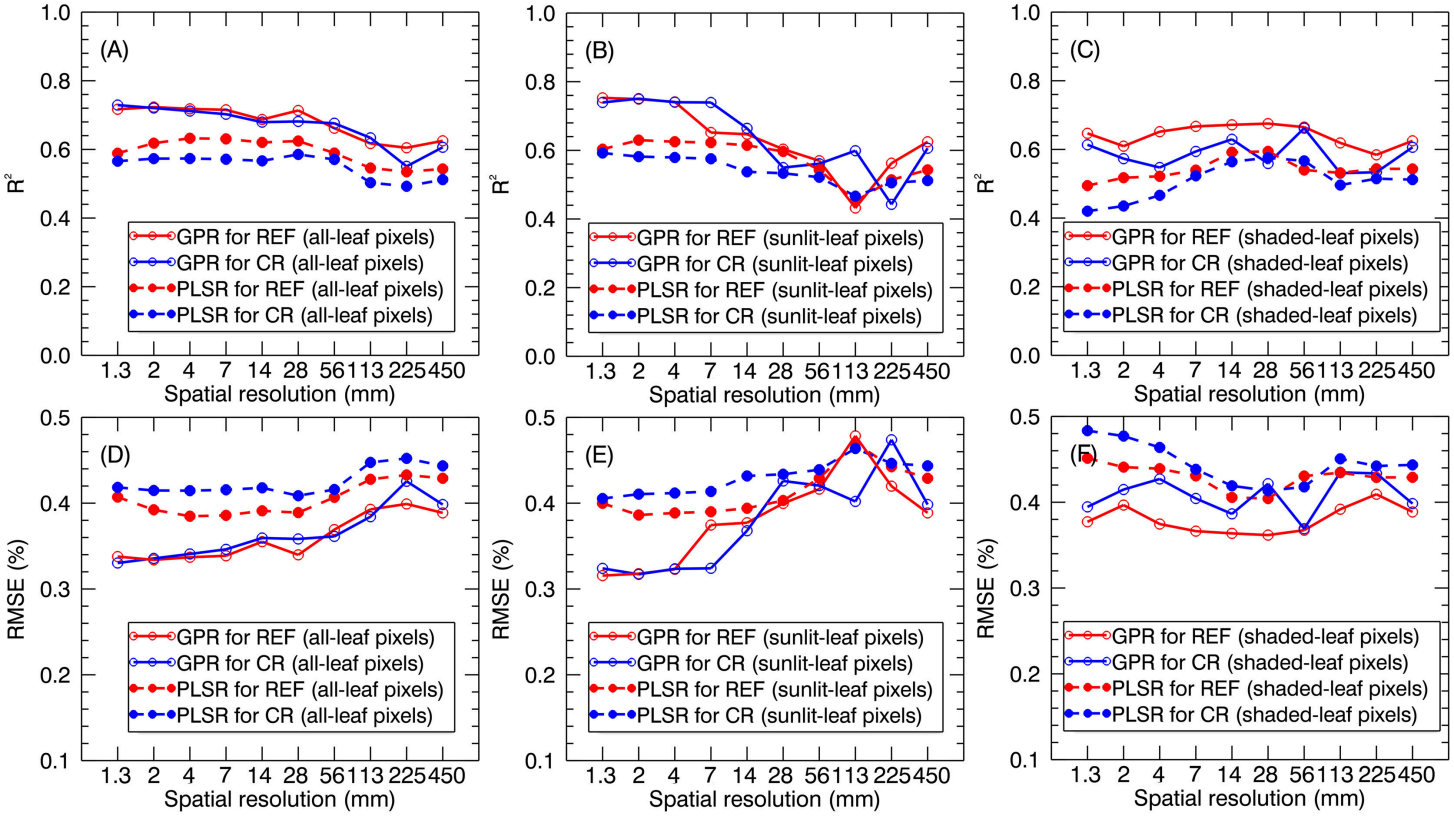
LNC prediction accuracies for GPR and PLSR using reflectance spectra (REF) and continuum-removed spectra (CR) derived from all-leaf pixels **(A,D)**, sunlit-leaf pixels **(B,E)** and shaded-leaf pixels **(C,F)**.

GPR generally exhibited higher *R*^2^ and lower RMSE values than PLSR regardless of spectral information derived from all-leaf pixels, sunlit-leaf pixels and shaded-leaf pixels (Figure 9). With regard to reflectance spectra and continuum-removed spectra, the former generally showed better predictive performances than the latter for PLSR but similar accuracies for GPR. Specifically, the reflectance spectra of shaded-leaf pixels exhibited higher *R*^2^ as compared to the continuum-removed spectra of shaded-leaf pixels when performing GPR.

**Figure 10:**
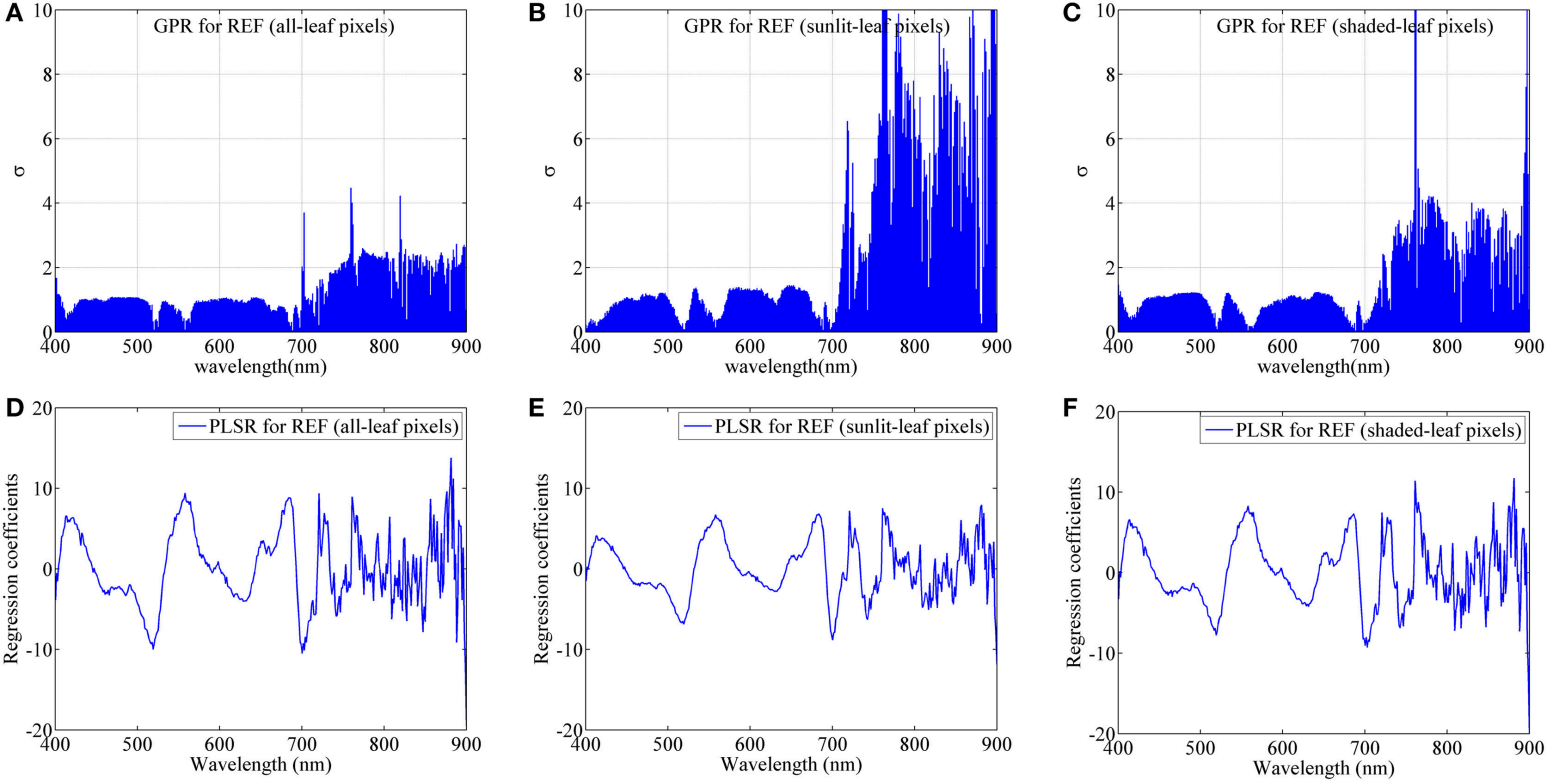
The dedicated parameter σ of each band for GPR and the regression coefficient of each band for PLSR using average reflectance spectra (REF) of all-leaf pixels **(A,D)**, sunlit-leaf pixels **(B,E)** and shaded-leaf pixels **(C,F)** from imaging data with 28 mm spatial resolution.

Figure 10 shows the band-by-band values of parameter σ for GPR models and regression coefficients for PLSR models from degraded imaging data with 28 mm spatial resolution, which is optimal for generating stable predictive performance. In the case of GPR model, the band with the lowest σ represents the most contribution to the regression model. As shown in Figure 10, most contributing bands were located around the red region (680 nm), the red-edge region (700 nm), the blue region (420 nm) and the green region (520 nm, 560 nm) regardless of leaf pixel types. Specifically, all-leaf pixels generally exhibited lower σ-values than sunlit- and shaded-leaf pixels over all the wavelengths. In contrast, the highest σ-values were observed in the near-infrared shoulder region for sunlit leaves.

For PLSR models, the importance of each band was evaluated with the coefficient values. The important bands (coefficients in absolute value) for PLSR models were found around the similar region as the GPR models. The absolute coefficient values for all-leaf pixels were generally higher than sunlit- and shaded-leaf pixels. However, there were no substantial differences in the coefficients of PLSR models among three types of leaf pixels in the near-infrared shoulder region.

## Discussion

We investigated the LNC~VI models and two multivariate regression models (i.e., PLSR and GPR) throughout the entire growing season of rice using multi-scale imaging spectroscopy data. To the best of our knowledge, this represents the first attempt to date to estimate rice LNC in paddy fields with near-ground hyperspectral imaging data.

### Comparison of the relationships with LNC between all-leaf derived VIs and whole-image derived VIs

Our results demonstrated that the all-leaf derived VIs generally correlated better with LNC than whole-image derived VIs (Table 4) during individual growth stages before booting, especially for the early tillering stage. This is consistent with previous studies on wheat (Vigneau et al., 2011) and sugar beet (Jay et al., 2014), which reported better predictive performances of LNC before canopy closure using the pure-pixel leaf spectra derived from field imaging spectrometer data. This is an important finding because optimal N fertilizer application depends heavily on the determination of crop LNC for improving the yield formation during early growth stages (Nguyen and Lee, 2006; Eitel et al., 2011; Yu et al., 2013; Tian et al., 2014). The lower correlations of LNC with VIs observed for whole-image pixels were mainly attributed to the confounding effects of soil, water or duckweed background interferences (Yu et al., 2013; Sun et al., 2017) present in the field of view. In this regard, the advantage of using field imaging spectrometers is the ability that enables us to extract the pure leaf pixels for directly monitoring leaf N status.

After the booting stage, rice canopy closure occurred in most field plots and the background materials were less exposed. This might lead to closer correlations with LNC for whole-image derived VIs (Table 4). However, all-leaf derived VIs still exhibited higher correlations with LNC than whole-image derived VIs for the stage group of reproductive stages (Table 4). These results represent a great potential for assessing rice N status during late growth stages to improve the prediction of grain quality (Yu et al., 2013).

### The impact of rice growth stage on LNC~VI models

A few studies have found that the relationships between VIs and LNC for early growth stages of paddy rice are different from those for later stages (Xue et al., 2004; Yu et al., 2013 and Tian et al., 2014), which was partly due to the difficulty in removing the confounding effects of background materials on the reflectance spectra of the whole canopy. Xue et al. (2004) demonstrated that LNC was related to VIs for each single stage instead of for all growth stages. Our results confirmed the influence of growth stages on the consistency of the LNC~VI relationships across growth stages (Xue et al., 2004 and Yu et al., 2013). Specifically, stage-specific regression models were found for the three early stages (i.e., early tillering, late tillering, and jointing) before booting. In contrast, the regression models for all the reproductive stages could be grouped into a single model. This is probably because rice crops usually uptake and accumulate N in leaves and stems in the vegetative period with the rate of leaf biomass accumulation exceeding the rate of N uptake (i.e., N dilution effect of growth with substantial decreases of LNC) (Sheehy et al., 1998; Yang et al., 2014). During vegetative stages, the performance of LNC~VI models were affected not only by the biomass or LAI, but also by the underlying water reflection in the paddy rice field (Mistele and Schmidhalter, 2008; Zhou et al., 2014). In contrast, these influences were reduced during reproductive stages because of relatively stable leaf biomass and less photon interaction with the underlying water background when the canopy closure occurred in most plots (Yu et al., 2013). In addition, the LNC in crops is often estimated from spectral data based on the relationship between LNC and leaf chlorophyll content (Schlemmer et al., 2013). However, this relationship would not hold the same for the whole season (Wang et al., 2014). For example, the distinct LNC~CI_Red−edge_ model for the early tillering stage were probably attributed to the lower values of leaf chlorophyll content but higher values of LNC (Wang et al., 2014). Although leaf or canopy chlorophyll content could be estimated with VI-based global models (Zhou et al., 2017), the estimation of LNC in crops was still performed with stage-specific models.

### Interpretation of the impacts of spatial resolution on the stage-specific LNC~VI relationships

Our results demonstrated that the correlations of VIs with LNC generally decreased as the spatial resolution degraded for each specific stage (or stage-group). This is mainly due to the background influence in mixed pixels. There were more difficulties in the discrimination of vegetation and background pixels from coarser spatial resolution imageries. This pattern was in agreement with the previous studies of resolution effects on the relationships between VIs and leaf traits such as the chlorophyll content (Jay et al., 2017). In particular, most ρ^2^-values remained stable when the resolution degraded from 1.3 to 14 mm for the early tillering stage but from 1.3 mm to a coarser resolution of 56 mm for the other stages (Figure 8). The main reason could be the fact that the green fraction values (average value of 0.15 for all the plots) for the early tillering stage were much lower than those for the other stages (Supplementary Figure 1). This would lead to more background influence in mixed pixels and more difficulties in the separation of vegetation and non-vegetation background pixels when using coarser images. Thus, the optimal resolution (14 mm) for the early tillering stage was much finer than other stages. In this regard, we suggested using finer resolution imageries (finer than 14 mm) at the early tillering stage of paddy rice.

The stability in the correlation with LNC over different spatial resolutions varied with leaf pixel types. The most stable performances for all-leaf pixels might be attributed to the more pixel numbers that representing more nitrogen-related spectral information as compared to sunlit- and shaded-leaf pixels. Specifically, all-leaf derived MTCI exhibited closet ρ^2^ between different spatial resolutions at specific stages. Given that MTCI was originally developed as an indicator of canopy chlorophyll content from low-resolution satellite sensors (Dash and Curran, 2004), the good performances for MTCI might be attributed to their relatively stable sensitivity with nitrogen-related leaf traits (e.g., leaf chlorophyll and nitrogen content) regardless of using high resolution data (Haboudane et al., 2008; Hunt et al., 2013; Jay et al., 2017) and low resolution data (Dash and Curran, 2004; Jay et al., 2017). Similar to the findings in Zhou et al. (2017), the results obtained here also exhibited higher correlations with nitrogen-related leaf traits for shaded-leaf pixels as compared to sunlit-leaf pixels when using finer resolution data. This could be explained by the fact that the multiple scattering among a larger fraction of shaded leaves enhances the apparent absorption features of leaves and leads to extended optical lengths with a not weak signal (Zhou et al., 2017). Furthermore, the specular reflection from sunlit leaves could negatively influence the spectroscopic estimation of LNC (Vigneau et al., 2011). The influence of specular reflection for sunlit-leaf pixels could be the main driver of the weaker relationships between VIs and LNC while using the images with finer spatial resolutions, especailly for the late tillering stage under strong illumination conditions (Figure 8). The spatial heterogeneity increased with the degradation of spatial resolution. When using the coarser images, the stronger correlations of sunlit-leaf derived VIs with LNC may be explained by the fact that the classified sunlit-leaf pixels were mixed with shaded-leaf pixels. However, the correlations could also decrease when using the spatial resolution coarser than 113 mm due to the confounding effect of the background materials.

Regarding the best cases for each specific stage, TCARI/OSAVI was highlighted as the optimal VI derived from finer resolution data for the early and late tillering stage but derived from coarser resolution data for the jointing stage. TCARI/OSAVI was developed to predict crop chlorophyll content from remote sensing data while minimizing the influence of leaf area index (i.e., canopy structure effects) and soil background effects (Haboudane et al., 2002). These advantages of TCARI/OSAVI could be the main driver of becoming the best performing VI for all the individual stages before booting when the environmental properties from low green fraction canopies adversely affects the spectroscopic estimation of LNC.

### Comparison of various approaches for spectral assessment of LNC

The use of 2~4 band VIs often constrains the regression analysis and cannot overcome the N dilution effect to build a global LNC~VI model for all the growth stages of paddy rice (Yu et al., 2013). In contrast, the application of the PLSR and GPR models both allows quantitative evaluation of leaf N status over rice canopies throughout the entire growing season (Figure 9). In contrast to the LNC-VI models, the PLSR and GPR models generated better predictive performances when using the spectra of sunlit-leaf pixels derived from the finer resolution images. This could be explained by the better ability of the multivariate regression methods (e.g., GPR and PLSR) to reduce the addictive effects caused by the specular reflection (Verrelst et al., 2012). Similar to the patterns observed for LNC~VI models, all-leaf pixels also exhibited more stable performances than sunlit- and shaded-leaf pixels when using PLSR and GPR models. The optimal spatial resolution was 28 mm since the predictive accuracies kept stable from 1.3 to 28 mm and then gradually decreased. This resolution was close to the optimal resolution of 35 mm found in Jay et al. (2017) for predicting leaf chlorophyll content in sugar beet canopies. This indicates that a compromise could be made around 28 mm to obtain a high sensitivity to LNC variations while minimizing background effects on the spectroscopic estimation of LNC.

As compared to PLSR, the better predictive performances for GPR might be because GPR can track both signal and noise properties and thus can partly overcome the blackbox problem by providing a ranking of both features and samples for a specific task (Verrelst et al., 2012, 2013). While using the continuum-removed spectra instead of reflectance spectra as the input spectra in the GPR and PLSR models, the chlorophyll absorption feature in the red region could be the main driver of the comparable or slightly worse performance. Specially, the most contributing band was located at the red spectral region around 680 nm (Figure 10), which is in accordance with the chlorophyll absorption peak (Inoue et al., 2012). The spectral areas close to the chlorophyll absorption peak in the blue spectral region (420 nm) were also found to be important for the GPR model. The other contributing bands were located at the red-edge region (700 nm) and the green region (520 and 560 nm). These two regions have been widely proved to be closely related with chlorophyll content and commonly used in the indices for the assessment of chlorophyll content such as CI_Red−edge_ and CI_Green_. It is worth noting that the NIR shoulder region was considered substantially less important for the GPR models based on sunlit-leaf pixels as compared to those based on shaded-leaf pixels. This could be explained by the fact that the NIR reflectance spectra of sunlit leaves were less influenced by the canopy structure parameters (Vigneau et al., 2011; Jay et al., 2017). In contrast, the NIR reflectance spectra of shaded leaves is not only related to biochemical parameters but is also governed by the variation in the canopy structural parameters. These structural variables could influence the multi-scattering within rice canopies and thus particularly affect the reflectance of shaded leaves in the NIR region (Verrelst et al., 2012; Zhou et al., 2017).

### Opportunities and limitations

Assessing the predictive performances of different methods showed that GPR models provided a better approach for estimating rice LNC at the field scale. This makes it possible to assess N status of individual plants and to benefit the spatially variable management of N throughout the entire growing season of paddy rice. In the case of rice crops, similar accuracies were obtained for all the resolutions finer than 28 mm, but the accuracies decreased for coarser resolutions. With the development of low-altitude unmanned aerial vehicle (UAV) systems, the combinations between hyperspectral cameras and UAVs are now becoming efficient and affordable and enable the acquisition of image measurements to a larger area with high spatial (e.g., 28 mm) and spectral resolutions simultaneously (Zarco-Tejada et al., 2012; Jay et al., 2017). In particular, the measurements of about 3 cm spatial resolution imageries can offer a good compromise between accuracy and efficiency for estimating LNC of paddy rice throughout the entire growing season from an appropriate flight altitude. This provides a practical approach for timely monitoring LNC of rice crops at critical growth stages.

Good robustness performances of LNC prediction are expected for paddy rice crops because the results in this study are derived from a combination of years, rice cultivars, planting densities, and N rates. However, the retrieval of whole canopy LNC could be further improved by using multi-angle observation data or three-dimensional reconstruction data (Jay et al., 2015) because of their better abilities to assess the N status of rice in middle and bottom layers as compared to using only the nadir viewing data (Li et al., 2013; He et al., 2016). Furthermore, various plant types (i.e., spread type, semi-spread type, or erect type) of rice cultivars can affect leaf surface effects, canopy structural properties, and N uptake processes (Li et al., 2013). In this regard, more genotypes should be included in our future work.

## Conclusions

We report on the use of near-ground imaging spectroscopy data to estimate LNC of rice crops throughout the entire growing season from 2-year experiments. Three group of VIs (a total of 16 indices) commonly used for the estimation of LNC and two multivariate methods (PLSR and GPR) were evaluated with spectral datasets of three types of leaf pixels (sunlit-, shaded-, and all-leaf) derived from millimeter to centimeter-scale imageries. The most important conclusions that derived from this study were as follows:

CI_Red−edge_, MTCI and TCARI/OSAVI were selected as the representative VIs from each group of VIs. The prediction of LNC needed stage-specific LNC~VI models before booting but could be performed with a single model across the stages after booting.

All-leaf derived VIs achieved more stable relationships with LNC over different spatial resolutions than sunlit- and shaded-leaf derived VIs. In particular, similar results were obtained from all the resolutions finer than 14 mm for the early tillering stage but from all the resolutions finer than 56 mm for the other stages.

TCARI/OSAVI performed the best for all the individual stages before booting. For the the reproductive stages, the best model was observed for all-leaf derived MTCI at 28 mm spatial resolution.

Both of the GPR and PLSR methods successfully performed the global predictions of LNC throughout the entire growing season. However, the GPR method generally achieved better predictive performances than the PLSR method.

Using the spectra of all-leaf pixels as the input variables in the PLSR and GPR models achieved more stable performances as compared to using the spectra of sunlit- and shaded-leaf pixels. The optimal spatial resolution was 28 mm since similar results were observed from 1.3 to 28 mm but deteriorated for coarser resolutions.

The approach of using GPR in this study take advantage of high spatial and spectral resolution to improve the estimation of LNC in paddy rice canopies as compared to commonly used VIs and PLSR method. Specifically, the use of reflectance spectra derived from all-leaf pixels at the spatial resolution of about 3 cm based on UAV observations is promising to accurately estimate LNC and also be useful for spatially variable applications of N fertilizers.

## Author contributions

KZ, TC, YZ, and WC conceived the idea and led the study design. KZ performed experiments with support by HZ; KZ, TC, and SU edited the manuscript; XY and YT assisted with study design and experiments. All authors read and approved the final manuscript.

### Conflict of interest statement

The authors declare that the research was conducted in the absence of any commercial or financial relationships that could be construed as a potential conflict of interest.

## Supplementary material

Supplementary Figure 1 Green fraction values (mean ± std) for individual growth stages. ET, early tillering stage; LT, late tillering stage; JT, jointing stage; BT, booting stage; HD, heading stage; FL, filling stage.

## Abbreviations

CI_Red−edge_: red edge chlorophyll index
EVI: enhanced vegetation index
GPR: Gaussian Process regression
LNC: leaf nitrogen concentration (%)
MTCI: MERIS terrestrial chlorophyll index
OSAVI: optimized soil-adjusted vegetation index
PLSR: partial least squares regression
PRI: photochemical reflectance index
TCARI: transformed chlorophyll absorption reflectance index
UAV: unmanned aerial vehicle
VIs: vegetation indices.
