# Supplementary figures and images for: Assessing the Impact of Spatial Resolution on the Estimation of Leaf Nitrogen Concentration Over the Full Season of Paddy Rice Using Near-Surface Imaging Spectroscopy Data
Source: Front Plant Sci. 2018 Jul 5;9:964. doi: 10.3389/fpls.2018.00964 (PMC6041568; doi:10.3389/fpls.2018.00964)

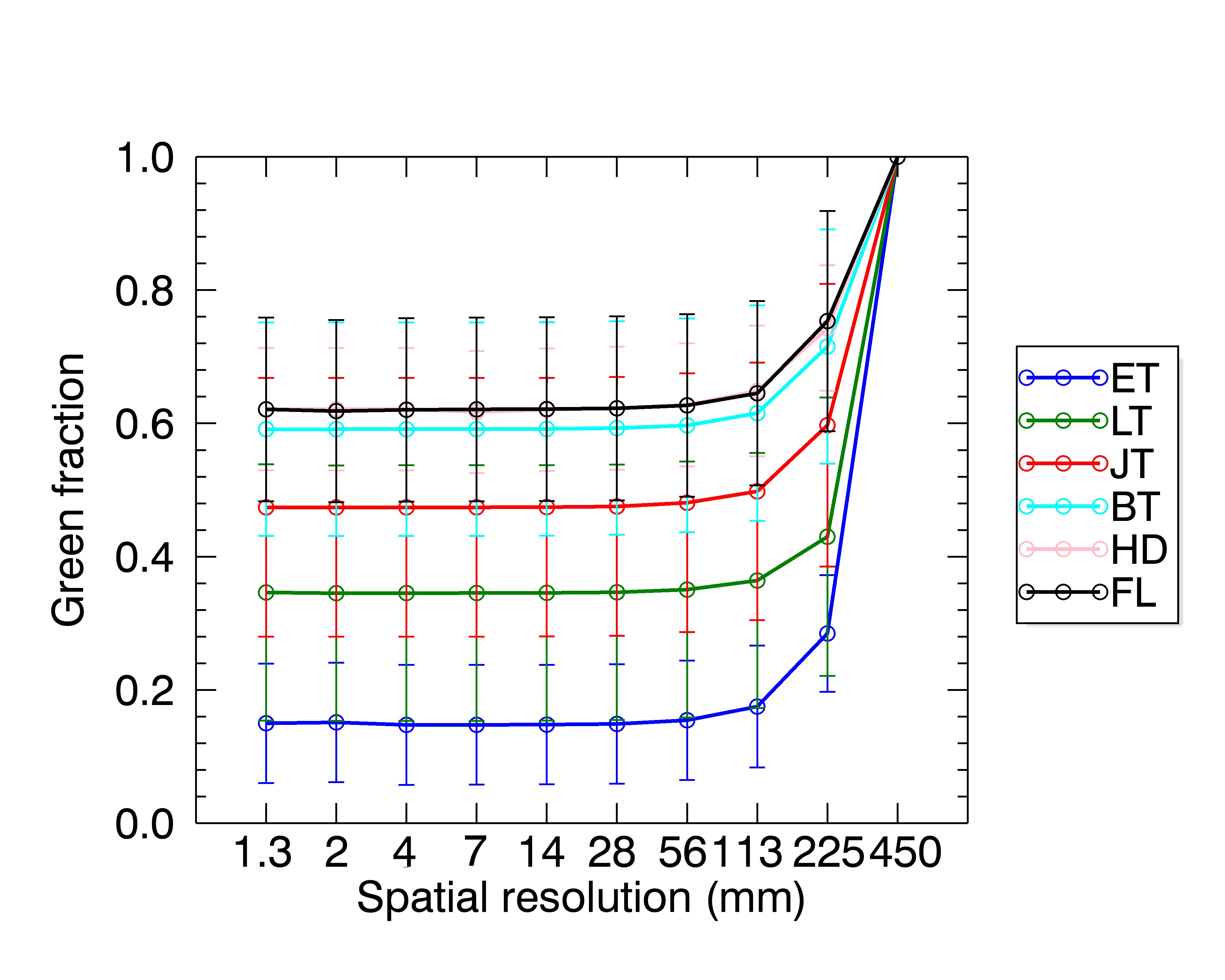

Supplement: Supplementary Figure 1 — Green fraction values (mean ± std) for individual growth stages. ET, early tillering stage; LT, late tillering stage; JT, jointing stage; BT, booting stage; HD, heading stage; FL, filling stage. [file Image_1.TIF]
